# High-Efficiency Expression and Purification of DNAJB6b Based on the pH-Modulation of Solubility and Denaturant-Modulation of Size

**DOI:** 10.3390/molecules27020418

**Published:** 2022-01-10

**Authors:** Sara Linse

**Affiliations:** Department of Biochemistry and Structural Biology, Lund University, P.O. Box 124, 221 00 Lund, Sweden; sara.linse@biochemistry.lu.se

**Keywords:** self-assembly, extraction, solubilization

## Abstract

The chaperone DNAJB6b delays amyloid formation by suppressing the nucleation of amyloid fibrils and increases the solubility of amyloid-prone proteins. These dual effects on kinetics and equilibrium are related to the unusually high chemical potential of DNAJB6b in solution. As a consequence, the chaperone alone forms highly polydisperse oligomers, whereas in a mixture with an amyloid-forming protein or peptide it may form co-aggregates to gain a reduced chemical potential, thus enabling the amyloid peptide to increase its chemical potential leading to enhanced solubility of the peptide. Understanding such action at the level of molecular driving forces and detailed structures requires access to highly pure and sequence homogeneous DNAJB6b with no sequence extension. We therefore outline here an expression and purification protocol of the protein “as is” with no tags leading to very high levels of pure protein based on its physicochemical properties, including size and charge. The versatility of the protocol is demonstrated through the expression of an isotope labelled protein and seven variants, and the purification of three of these. The activity of the protein is bench-marked using aggregation assays. Two of the variants are used to produce a palette of fluorescent DNAJB6b labelled at an engineered N- or C-terminal cysteine.

## 1. Introduction

DNAJB6 belongs to a group of chaperons, also called J-domain proteins or HSP40, characterized by the presence of a J-domain [1,2,3]. Two member proteins, DNAJB6b and DNAJB8, are recognized as potent inhibitors of amyloid formation in vitro and in vivo by a range of proteins and peptides including IAPP from diabetes type II [4], poly-Q peptides from Huntington’s disease [1,5,6,7,8,9], α-synuclein from Parkinson’s disease [10,11,12] and amyloid β peptide from Alzheimer’s disease [13,14,15]. While amyloid is recognized as a generic state that most peptides/proteins or parts thereof may attain under some solution conditions [16], DNAJB6b and DNAJB8 are thus promiscuous amyloid formation inhibitors acting on a wide range of proteins.

The current study concerns the 241-residue DNAJB6b isoform, which is found in the cytosol and nucleus of cells. The DNAJB6a isoform is only found in the nucleus, differs at 10 residue positions in the C-terminal domain, and contains a nuclear localization signal in a C-terminal extension. Familial mutations in the DNAJB6 gene are connected to limb-girdle muscular dystrophy [17,18,19]. As reviewed [20], most of the familial mutations in DNAJB6 are found in the region including amino acids 89–100.

In a solution, depending on the total protein concentration, DNAJB6b may form large and highly polydisperse oligomers [6]. The three-dimensional structure of the protein has been studied using nuclear magnetic resonance spectroscopy yielding high-resolution structural models of the N-terminal J-domain rich in α -helices as well as the C-terminal domain rich in β-sheet [21]. However, the relative orientation of the two globular domains remains elusive and is most likely highly variable because the domains are connected by a very long linker whose composition is of low complexity. A model for a dimeric form of DNAJB6b based on covalent cross-linking data has been presented [22].

The current study is motivated by the very potent roles of DNAJB6b as an inhibitor of amyloid formation and an enhancer of amyloid solubility [14]. The retardation of amyloid formation is understood as an interaction with pre-fibrillar species hindering their nucleation into a fibrillar structure [13,14,15], a mode of action shared with several anti-amyloid chaperones including, for example, clusterin [23] and HSP70 [24].

The increase in solubility, i.e., the monomer concentration in equilibrium with aggregates of Aβ42, amounts to an impressive factor of ca. 500 in the presence of a 0.1:1 molar ratio of DNAJB6b:Aβ42. This can be reconciled with the formation of co-aggregates between Aβ42 and DNAJB6b with altered structure compared to pure Aβ42 fibrils [25]. This is a consequence of the second law of thermodynamics; if a molecule is present in co-existing phases at equilibrium, the chemical potential of the molecule must be the same in all these phases [26,27]. Thus, if the same peptide, in this case Aβ42, displays a difference in solubility in samples with a different composition, i.e., the chemical potential of the free monomer is not the same in these samples, then it follows that the structure of the aggregates in these samples must be different enough that the chemical potential of the monomers in the aggregates is altered. The unfavorable increase in the chemical potential of Aβ42 in the co-aggregates must be compensated by a reduction in the chemical potential of the chaperone in the co-aggregates relative to pure chaperone assemblies, such that the free energy of the system as a whole is lowered upon formation of the co-aggregates. This further implies that the chaperone on its own has a high chemical potential, the molecular origin of which remains elusive.

Biophysical and structural investigations of DNAJB6b alone and in co-aggregates with amyloid peptides may enhance our understanding of the molecular driving forces and structural features behind both the co-assembly process and the unusually high chemical potential of DNAJB6b. This will require highly pure and homogeneous protein devoid of tags or other sequence extensions to ensure that the physicochemical properties are not altered. The most straightforward way to achieve this is to express the proteins “as is” with their native sequence and to develop a purification protocol based on their physico-chemical properties, thus eliminating the need for expensive proteases and post-cleavage steps.

Here we develop facile and scalable protocols for the expression and purification of DNAJB6b “as is”, i.e., devoid of tags or other sequence additions. For the initial isolation from an *E. coli* cell pellet, we utilize the large difference in DNAJB6b solubility depending on the solution pH. For the subsequent purification of the protein, we utilize the large difference in size depending on the presence of a moderate concentration of denaturant (1.5–2.0 M GuHCl). Consecutive size-exclusion steps in the presence and absence of denaturant are thus used to remove any remaining large and small contaminants, respectively. The robustness of the protocol is validated through activity assays and through the expression and purification of sequence variants, two of which facilitate the production of a palette of fluorescent DNAJB6b with different excitation and emission wavelengths for spectroscopic and microscopic studies.

## 2. Results

### 2.1. Expression

**Figure 1:**
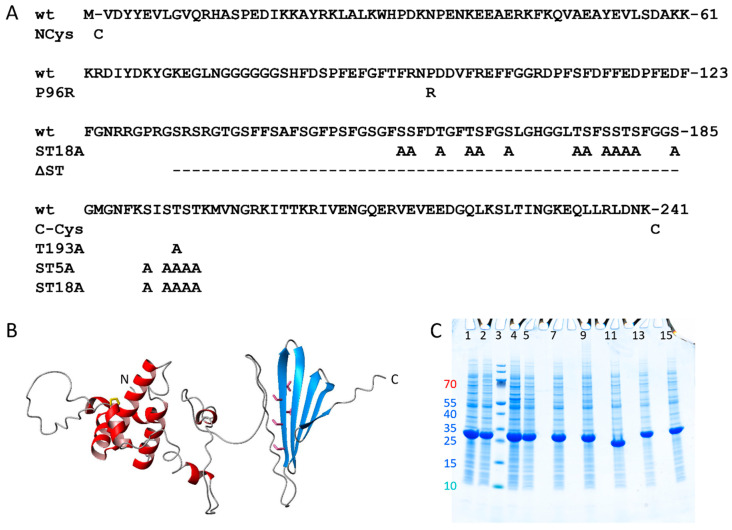
Expression of DNAJB6b wt and mutants. (**A**) Sequence of DNAJB6b wt and the substitutions made in the mutants of this study. (**B**) Prediction of DNAJB6b wt using Alpha-fold2 [28] with the J-domain shown in red, the C-terminal domain in blue, Pro96 in yellow, Ser190, Ser192, Thr193, Ser194 and Thr195 in pink. (**C**) Whole *E. coli* extract after expression of DNAJB6b wt and mutants in rich medium (lane 1, 4, 5, 7, 9, 11, 13 and 15) or M9 minimal medium (lane 2). Lane 1, 2 = wt DNAJB6b. Lane 3 = M_w_ standard with the M_w_ of the 7 smallest proteins given to the left of lane 1. Lane 4 = NCys- DNAJB6b. Lane 5 = CCys- DNAJB6b. Lane 7 = DNAJB6b-ST5A. Lane 9 = DNAJB6b -ST18A. Lane 11 = DNAJB6b-ΔST. Lane 13 = DNAJB6b -P96R. Lane 13 = DNAJB6b -T193A. Cell pellets from 1 mL were dissolved in 800 µL of 8 M urea, pH 8.0, mixed 1:1 with SDS loading buffer and 3 µL loaded per lane. The quantity loaded in each lane thus corresponds to cells from 2 µL culture.

The protein was expressed in *Escherichia coli* BL21 DE3 pLysS star “as is”, i.e., with no tags or sequence extensions, from a synthetic gene with *E. coli*-preferred codons to yield the human DNAJB6b protein with the amino acid sequence given in Figure 1A, and the predicted 3D-structure in Figure 1B. As shown in Figure 1C, the expression level of wt DNAJB6b is around 1.5 g/L in rich auto-induction medium and only slightly lower in M9 minimal medium used for ^13^C and ^15^N labelling.

### 2.2. Isolation

**Figure 2:**
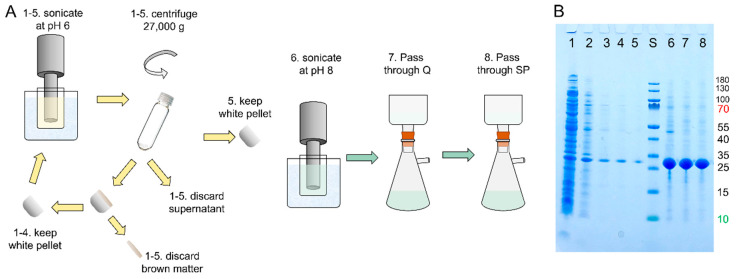
Initial isolation of DNAJB6b from *E. coli* cell pellet. (**A**) Outline of the isolation steps. 1–5. Sonication in 20 mM MES, pH 6.0 (supernatant after centrifugation). 6. Sonication in 10 mM Tris/HCL, 1 mM EDTA, pH 8.0. 7. Passage through Q-sepharose big beads. 8. Passage through SP sepharose HP. (**B**) SDS PAGE on a 10–20% polyacrylamide gel with lane 1–5 loaded with sonicate 1–5, lane S with M_w_ standard with sizes given to the right of the gel, lane 6 with sonicate 6, lane 7 with the Q flow-through, and lane 8 with the SP sepharose flow-through. The total time required for step 1–8 is ca. 2 h. The flow-through of SP sepharose (lane 8) was used for purification of the protein using ammonium sulphate precipitation and size-exclusion chromatography (see Figure 3).

The purification protocol starts with isolation of the protein from *E. coli* using repeated sonication and centrifugation at pH 6.0, conditions under which the protein forms a white precipitate and *E. coli* proteins are removed in the supernatant (Figure 2). At each sonication step, the pellet, after centrifugation, has a white-grey body covered with brown matter. The brown matter is scraped off using a spatula and discarded before the next round of sonication. After five rounds of sonication at pH 6.0, centrifugation and scraping, the pellet is sonicated at pH 8.0, conditions under which DNAJB6 rapidly goes into solution (Figure 2). Charged contaminants are then removed by passing this final sonicate through an anion exchange resin (Q sepharose big beads) and then through a cation exchange resin (SP sepharose HP) (Figure 2).

The total isolation procedure takes ca. 2 h from the removal of the cell pellet from the freezer to aliquoting the flow-through from the SP sepharose. Using the cell pellet from 0.25 L, the procedure results in a 200 mL solution with a total of ca. 300 mg semi-pure DNAJB6b, thus corresponding to ca. 1.2 g protein per liter of culture (Table 1).

### 2.3. Ammonium Sulphate Precipitation

Further purification is achieved using ammonium sulfate (AMS) precipitation to remove non-protein contaminants as well as some of the protein contaminants. After finding the relevant AMS concentration range using small volume samples and SDS PAGE (Supplementary Figure S1), bulk precipitation was performed for 25 mL aliquots and brought forward to the next step.

The time consumption for this step is ca. 30 min, and the losses are ca. 25% meaning that after this step, there remains ca. 900 mg protein per liter of culture.

### 2.4. Size Exclusion Chromatography

**Figure 3:**
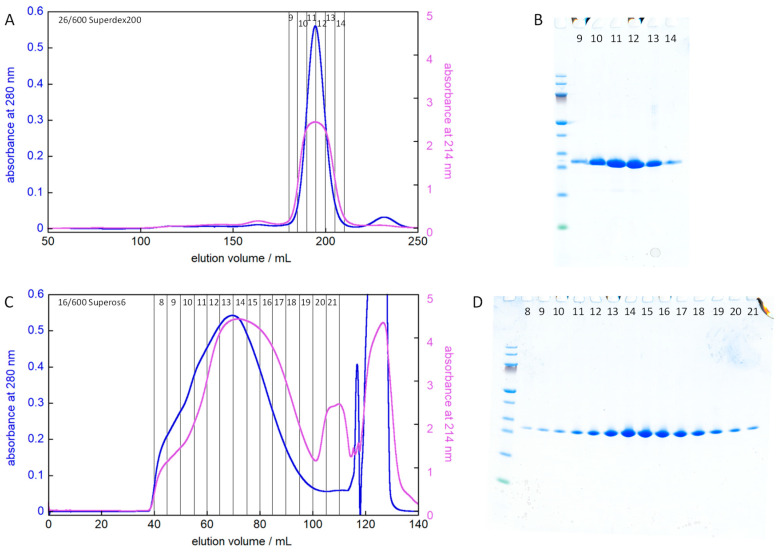
Purification of DNAJB6b using size exclusion chromatograph. (**A**) An aliquot from the flow-through of SP sepharose (see lane 8 in Figure 2) was precipitated by AMS and the 10–21% fraction dissolved in 10 mL 6 M GuHCl, 20 mM sodium phosphate, 0.2 mM EDTA, pH 8.0 and injected on a 26/600 Superdex200 column operated in 2 M GuHCl, 20 mM sodium phosphate, 0.2 mM EDTA, pH 8.0. (**B**) SDS PAGE of fractions 9–14 on a 10–20% polyacrylamide gel. Fraction 10–13 were concentrated and injected on a 16/600 Superose6 column. (**C**) The elution of the 16/600 Superos6 column operated in 20 mM sodium phosphate, 0.2 mM EDTA, pH 8.0. The injected sample was 5 mL of fractions 10–13 from panels A, B lyophilized down to 1/3 of the original volume, i.e., in 6 M GuHCl, 60 mM sodium phosphate, 0.6 mM EDTA, pH 8.0. (**D**) SDS PAGE of fractions 8–21 on a 10–20% polyacrylamide gel. In panels A and C, the absorbance at 280 and 214 nm are shown in blue and purple, respectively. Fractions 11–18 are kept for use in biophysical experiments.

Size-exclusion chromatography (SEC) was used to remove low and high Mw contaminants and to exchange the buffer to the one used in biophysical experiments (Figure 3). The first SEC step is performed with 2 M GuHCl to favor dissociation of DNAJB6 oligomers and using a Superdex200 resin (separation range 10–600 kDa) to remove large protein contaminants from the AMS precipitate, which was dissolved in 6 M GuHCl prior to injection. The second SEC step is performed under native conditions and using a Superos6 resin (separation range 5–5000 kDa) to remove lower Mw contaminants. Based on SDS PAGE analysis, this combination of SEC steps results in a highly pure protein.

The run time for the two SEC steps is ca. 100 and 80 min, respectively, but with the intervening lyophilization preferably run overnight. The loss per SEC step is ca. 25% meaning that after these final two steps, the total protocol results in ca. 600 mg ultra-pure protein per liter of culture (Table 1). The total operation time is ca. 5.5 h, plus the time required to run SDS PAGE.

### 2.5. Concentration Determination

**Figure 4:**
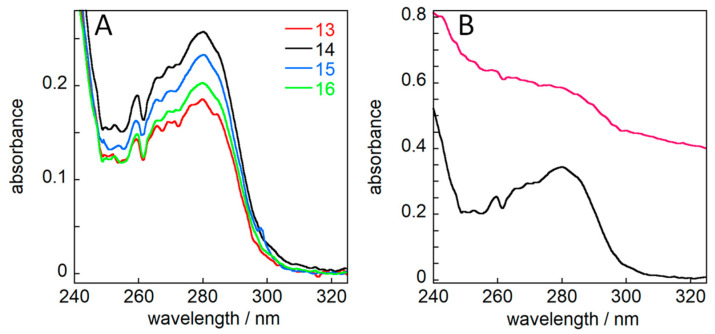
Concentration determination. (**A**) Absorbance spectra of fractions 13–16 from the elution of Superos6 as shown in Figure 3C,D. A small sample from each fraction was mixed 3:1 with 8 M GuHCl to bring the samples to 2 M GuHCl, which leads to the dissociation of the large polydisperse oligomers. (**B**) Absorbance spectrum of fraction 14 in buffer (pink), in which case the large polydisperse oligomers lead to significant light-scattering prohibiting concentration determination, and in 2 M GuHCl, which permits absorbance to be used for concentration determination.

DNAJB6b in solution exists as polydisperse oligomers that scatter light, which compromises concentration determination using absorbance. This is solved by diluting the samples 3:1 with 8 M GuHCl, yielding a 75% solution in 2 M GuHCl (Figure 4). Back-calculation to 100% gives the sample concentration of each fraction. Based on this information, the total yield after the second SEC step is 600 mg purified protein per liter of culture.

### 2.6. Robustness of Expression Protocol

The robustness of the tag-free expression system was evaluated for seven variants referred to as NCys with a cysteine residue added at the N-terminus, CCys, with a cysteine residue added at the C-terminus, STA5 with 5 alanine residues replacing Ser190, Ser192, Thr193, Ser194, and Thr195, ST18A with 18 different S -> A and T -> A substitutions, ΔST with a large part of the Ser/Thr-rich region deleted, P96R with an Arg residue replacing Pro96, and T193A with Thr193 changed to Ala. The amino acid substitutions in the variants are given in Figure 1. We find that all seven variants express to very high levels, similar to wt, and clearly dominate over all the *E. coli* proteins in the whole-cell extracts (Figure 1C).

### 2.7. Robustness of Purification Protocol

**Figure 5:**
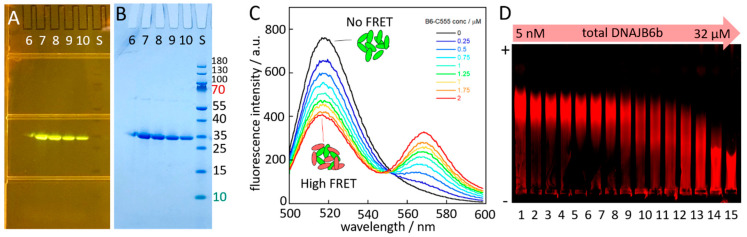
Fluorescent DNAJB6b. (**A**,**B**) Examples of SDS PAGE after labelling and SEC to remove free dye for DNAJB6b-CCys labelled with Alexa-488. (**A**)The gel imaged on a “dark reader” with blue excitation filter and orange emission filters. (**B**) The same gel photographed after staining with coomassie (quick stain). Panel (**C**) shows fluorescence emission spectra recorded for samples with 1 µM constant concentration of DNAJB6b-CCys-Alexa-488 and varying concentrations of DNAJB6b-CCys-Alexa-555: 0 (black), 0.25 (marine), 0.5 (blue), 0.75 (light blue), 1.0 (cyan), 1.25 (green), 1.5 (yellow), 1.75 (orange) and 2.0 µM (red). (**D**) Native gel electrophoresis. Agarose gel imaged on an IR fluorescence scanner. In each well is loaded 5 nM DNAJB6b-CCys-IR680 mixed with different concentrations of unlabeled DNAJB6b-wt to yield the following total concentrations of DNAJB6b: 5 nM (lane 1), 9 nM (2), 13 nM (3), 21 nM (4), 37 nM (5), 69 nM (6), 133 nM (7), 255 nM (8), 505 nM (9), 1.0 µM (10), 2.0 µM (11), 4.0 µM (12), 8.0 µM (13), 16.0 µM (14), 32 µM (15). The gel is oriented with the positive pole at the top of the image and the negative pole at the bottom.

The robustness of purification was evaluated for three variants, NCys, CCys, and STA5. STA5 was purified using the same protocol as for wt (Figure S1). NCys and CCys (Figure 5) were purified using the same protocol as for wt, except that 1 mM DTT was included in all buffers.

### 2.8. Activity of the Purified Protein

**Figure 6:**
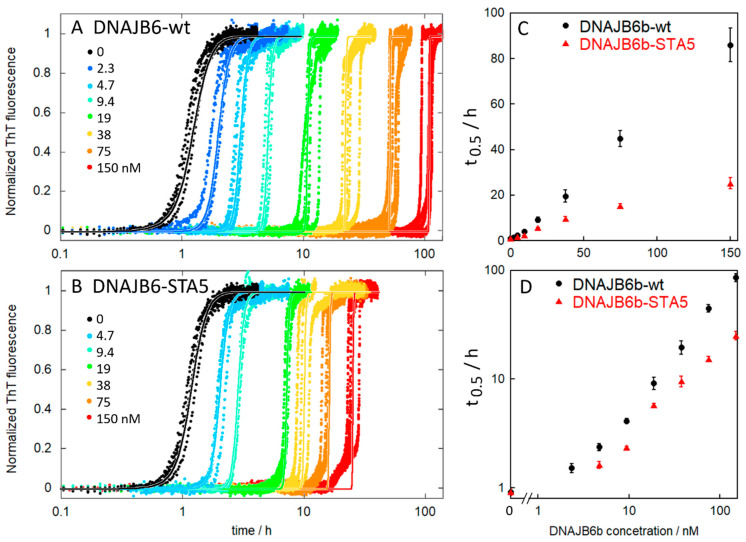
Aggregation kinetics. The activity of the purified proteins was validated by monitoring the fibril formation of 4 µM Aβ42 by ThT fluorescence in the absence (black) and presence (colors) of DNAJB6b-wt (**A**) or DNAJB6b-STA5 (**B**) at concentrations ranging from 2 to 150 nM, i.e., at molar ratios ranging from 0.0005 to 0.0375. The solid lines show fitted curves assuming inhibition of primary nucleation. The half times of aggregation as extracted from the data in panels A and B are shown as averages and standard deviations over 4 replicates for DNAJB6b-wt (black) and DNAJB6b-STA5 (red) with linear (**C**) and logarithmic (**D**) axes.

The activity of the DNAJB6b protein was investigated by monitoring the fibril formation of Aβ42 by ThT fluorescence in the absence and presence of the purified DNAJB6b wt or STA5 at a series of concentrations (Figure 6). The wt protein is a very potent inhibitor of Aβ42 aggregation, and we observe a substantial delay of the sigmoidal transition from 0.0005:1 molar ratio of DNAJB6b:Aβ42 and upwards. The data are well fitted by a model that includes primary nucleation, elongation, and secondary nucleation, keeping the rate constants for elongation and secondary nucleation as global parameters and the rate constants for primary nucleation as a local parameter specific to each DNAJB6b concentration (see solid lines in Figure 6). Inhibition of primary nucleation can thus explain all the data obtained in the presence of DNAJB6b. The data for the DNAJB6b-STA5 mutant are also well fitted assuming inhibition of primary nucleation, but the variant appears less effective than the wt (Figure 6). These findings are in agreement with those of earlier reports [13,14].

### 2.9. Fluorophore Labelling

Each of the cysteine containing constructs was labelled with a palette of fluorophores including Alexa488, Alexa555, Alexa647, and IRdye-680. DTT was first removed using SEC and maleimide dyes were added from stocks in DMSO, followed by a second SEC step to isolate the labelled protein and to remove the excess free dye (Figure 5).

### 2.10. Polydispersity

Depending on the total protein concentration, DNAJB6b may form large and highly polydisperse aggregates in solution [6]. This is evident from the SEC under non-denaturing conditions, in which case DNAJB6b elutes as a very broad peak from a superos6. This is also evident from native electrophoresis in an agarose gel of IR680-labelled DBAJB6b at a constant concentration of 5 nM with a variable concentration of unlabelled DBAJB6b (Figure 5).

## 3. Discussion

The results of the current study show that *E. coli* cells are highly tolerant to DNAJB6b expression. In the whole-cell extracts (Figure 1), the overexpressed chaperone clearly dominates over all the *E. coli* proteins, and this is observed for DNAJB6b wt as well as all seven mutants tested. This provides a very good starting state for isolation and purification of the proteins and relies on the use of synthetic genes with *E. coli* optimized codons in the expression plasmids on the DNAJB6b protein causing no impairment of *E. coli* growth.

Studies of protein biophysical properties require highly pure protein devoid of tags or other sequence extensions. The main advantage of the current expression protocol is that the production of tag-free proteins makes the following isolation and purification straightforward. No expensive proteases or post-cleavage steps are needed. Instead, the protocol is built on the physico-chemical properties of the protein in terms of charge and size and the modulation of these properties by pH and denaturant, respectively. We have expressed the protein “as is” with its native sequence and developed a purification protocol based on its physico-chemical properties.

**Figure 7:**
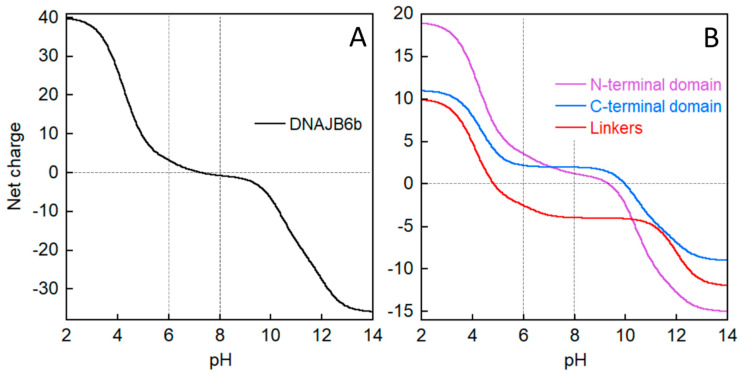
Net charge of DNAJB6b (**A**) and its parts (**B**) as a function of pH titration as calculated based on model compound pKa values [29].

In the initial isolation of the protein from *E. coli* cell pellet (Figure 2), we utilize the large difference in DNAJB6b solubility depending on pH. Intriguingly, DNAJB6b is highly soluble in the form of polydisperse oligomers at pH 8.0 and displays a minimum solubility around pH 6.0, although the isoelectric point is 7.3 if calculated from its amino acid sequence using model pKa values ([29]; Figure 7A). Precipitation at pH 6.0 likely results from titration of the N-terminal domain and the linker, because the C-terminal domain (residues 187-241) displays constant positive net charge at around +2 between pH 8.0 and 6.0 (Figure 7B). The net charge of the N-terminal domain changes from ca. +1.2 at pH 8.0 to ca. +3.6 at pH 6.0, while that of the linker changes from ca. −4.0 to −2.5 (Figure 7B). The low solubility at pH 6.0 allows the initial isolation protocol to use repeated sonication at pH 6.0 and removal of soluble *E. coli* proteins by decanting the supernatant over the precipitate and by removal of the less dense precipitate by scraping away brown matter from the top of the white DNAJB6b pellet. This scraping procedure sacrifices yield for purity, but the final yield is very high anyway given the extreme expression level and for the subsequent biophysical studies it is much more important to focus on achieving as high purity as possible. The isolation procedure ends with solubilization of the protein through sonication at pH 8.0 and removal of charged contaminants through stepwise passage of the sonicate through anion and cation exchange resins. Here, it is fortuitous that DNAJB6b carries such a low net negative charge of ca. −0.8 that it is not retained by anion nor cation exchange resins.

Because DNAJB6 forms highly polydisperse oligomers, it elutes very broadly during size exclusion chromatography and is difficult to separate from impurities of all sizes except possibly those smaller than the DNAJB6b monomer. To circumvent this problem, we used a first SEC step in the presence of enough denaturant that the oligomers dissociate and DNAJB6b elutes as a relatively narrow peak on a Superdex200 column relatively late in the chromatogram meaning high Mw protein contaminants are removed by this step. This first SEC step was preceded by ammonium sulphate (AMS) precipitation to remove non-protein contaminants as well as some protein contaminants. The second SEC step on a superos6 column serves to exchange the buffer to the one used in the following biophysical experiments and to remove any remaining small proteins. In this step, the protein is injected denatured in 6 M GuHCl but folds and oligomerizes as it elutes ahead of the denaturant.

The result is highly pure DNAJB6b amenable for detailed studies of its biophysical properties, such as structure and dynamics, stability, polydispersity, exchange rates, etc. aiming at an understanding of the molecular origin of its unusually high chemical potential [26] and the driving force for its co-assembly with amyloid peptides. The ease of adaption to mutational and sequence length variants is another advantage of the protocol. NCys and CCys were produced to enable site-specific covalent attachment of fluorophores or nanogod labels to either terminus using maleimide chemistry. Although fluorophores should be used with care as an appendix the size of 7–10 residues with mixed non-polar and charged character is added to the protein under study, the possibility of including a small fraction of labelled DNAJB6b will enable the study of the biophysical properties of mostly unlabelled oligomers. STA5 and STA18 were chosen based on earlier work showing reduced inhibitory capacity [14]. ΔST was chosen based on earlier work showing reduced polydispersity and a larger fraction of monomeric protein for this variant [21]. P96R and T193A were based on the disease association of these mutants [20].

## 4. Methods

### 4.1. Gene Synthesis and Cloning

The protein was expressed in *Escherichia coli* BL21 DE3 PlyS star “as is”, i.e., with no tags or sequence extensions, from a synthetic gene with *E. coli*-preferred codons to yield the human DNAJB6b wt protein with the amino acid sequence as shown in Figure 1, as well as seven variants. The genes were cloned between NdeI and BamHI restriction sites in a Pet3a vector. The gene synthesis and cloning were purchased from Genscipt (Piscataway, NJ, USA).

### 4.2. Expression in Rich Medium

The plasmid was transformed into Ca^2+^ competent *E. coli* BL21 DE3 PLysS Star and spread on LB-agar plates with 50 mg/L ampicillin and 30 mg/L chloramphenicol. Single small and well-isolated colonies were used to inoculate 50 mL LB cultures with 50 mg/L ampicillin and 30 mg/L chloramphenicol, which were grown at 37 °C in 250 mL baffled flasks with 125 rpm orbital shaking for 8 h. The OD at 600 nm was measured and the equivalent of 0.5 mL at OD600 = 0.8 was transferred to 500 mL pre-warmed (37 °C) overnight express medium. One liter of medium was prepared mixing the following separately autoclaved solutions: (a) 1 mL 1 M MgSO_4_, (b) 50 mL“20 × 5052”: 5 g glycerol, 0.5 g glucose, 2 g α-lactose, 44.6 mL H_2_O, (c) 50 mL “20 × NPS”: 3.3 g (NH_4_)_2_SO_4_, 6.8 g KH_2_PO_4_, 7.1 g Na_2_HPO_4_, 45 mL H_2_O, and (d) 900 mL “1.1 × LB”: 10 g Bacto^TM^ tryptone, 5 g Bacto^TM^ yeast extract, 10 g, H_2_O up to 900 mL, and adding 50 mg/L ampicillin and 30 mg/L chloramphenicol from concentrated stocks. The overnight express cultures were grown in 2500 mL baffled flasks with 125 rpm orbital shaking for 15 h, after which the cells were harvested by centrifugation at 6000 rpm for 10 min in a JLA 8.1000 rotor.

### 4.3. Expression in M9 Minimal Medium with Isotope Labels

The plasmid was transformed into Ca^2+^ competent *E. coli* BL21 DE3 PLysS Star and spread on LB-agar plates with 50 mg/L ampicillin and 30 mg/L chloramphenicol. Single small and well-isolated colonies were used to inoculate 50 mL LB cultures with 50 mg/L ampicillin and 30 mg/L chloramphenicol, which were grown at 37 °C in 250 mL baffled flasks with 125 rpm orbital shaking for 14 h. 3 mL was transferred to 500 mL pre-warmed (37 °C) M9 medium. One liter of medium was prepared by mixing the following separately autoclaved solutions: (a) 980 mL M9 salt solution: 4.65 g Na_2_HPO_4_·2H_2_O, 3 g KH_2_PO_4_, 0.5 g NaCl, tap water up to 980 mL. (b) 10 mL of 200 g/L 13C-glucose, (c) 10 mL of 100 g/L ^15^NH_4_Cl, (d) 1 mL 1 M MgSO_4_, (e) 0.4 mL of 250 mM CaCl_2_ and the following sterile filtered solutions 0.6 mL of 30 mM FeCl_3_, (f) 1 mL of 1 mg/mL vitamin B1 and adding 50 mg/L ampicillin and 30 mg/L chloramphenicol from concentrated stocks. The cultures were grown in 2500 mL baffled flasks with 125 rpm orbital shaking until they reached an OD at 600 mm of 0.8, at which point 0.2 mM IPTG was added from a freshly prepared stock and the cells were harvested 5 h later by centrifugation at 6000 rpm for 10 min in a JLA 8.1000 rotor.

### 4.4. Purification Procedure

Step 1—sonication at pH 6.0. The pellet from 0.5 L culture was sonicated in 80 mL 20 mM MES, pH 6.0, in a glass beaker immersed in an ice-water slurry using a Soni-Prep 150 sonicator, large horn, 50% duty cycle, for three minutes. The slurry was centrifuged for 6 min at 4 °C at 18,000 rpm in a Beckman 25:50 rotor. The supernatant (lane 1 in Figure 2) was discarded and the top of the pellet was scraped with a spatula to remove brown matter. The pellet was re-sonicated four times in 80 mL 20 mM MES, pH 6.0, then centrifuged for 6 min at 4 °C at 18,000 rpm as above. After each centrifugation, the supernatant (lane 2–5 in Figure 2) was discarded and the top of the pellet was scraped with a spatula to remove brown matter, the amount of which significantly reduced after each sonication and centrifugation round.

Step 2—sonication at pH 8.0. The pellet after the fifth sonication above was sonicated and dispersed using a magnetic stir bar in a total of 200 mL 10 mM tris, 1 mM EDTA, pH 8.0, resulting in a clear solution (lane 6 in Figure 1).

Step 3—ion exchange removal of charged contaminants. The DNJAB6b solution after step 2 was first passed through 50 mL Q Sepharose big beads (anion exchange resin, lane 7 in Figure 2 shows the flow-through) and then through 25 mL SP HP resin (cation exchange resin, lane 7 in Figure 1 shows the flow-through).

Step 4—ammonium sulphate precipitation. The flow-through of the SP HP resin was mixed with 100% ammonium sulphate (AMS), pH 8.0 (equilibrated over ammonium sulphate salt at room temperature) at a 100:17.6 volume ratio resulting in 15% AMS, incubated for 10 min in an ice box, and centrifuged for 10 min at 8000 rpm in a JLA 8.1000 rotor. The supernatant was mixed with 100% AMS pH 8.0 at a 117.6:10.6 volume ratio, resulting in 22% AMS (the 15–22% fraction). The sample was incubated for 10 min in an ice box and centrifuged for 10 min at 8000 rpm in a JLA 8.1000 rotor.

Step 5—size exclusion chromatography. Each pellet from step 4 was dissolved in 6 M GuHCl and separated by size exclusion chromatography (SEC) on a 26/600 mm Superdex200 column (GE Healthcare) operated in 2 M GuHCl, 20 mM sodium phosphate, 0.2 mM EDTA, pH 8.0 (Figure 3A,B). Under these conditions, DNAJB6b elutes as a monomer or dimer. Fractions were analyzed using the absorbance at 214, 260, 280 nm and by SDS PAGE (10–20% gel from Novex in a Tris/Tricine buffer system). Fractions containing DNAJB6b were lyophilized to concentrate them three times, followed by SEC on a 16/600 mm Superose6 column (GE Healthcare) operated in 20 mM sodium phosphate, 0.2 mM EDTA, pH 8.0 (Figure 3C,D). Under these conditions, DNAJB6b elutes as highly polydisperse oligomers. Fractions were analyzed using the absorbance at 214, 260, 280 nm and by SDS PAGE (10–20% gel from Novex in a Tris/Tricine buffer system). Fractions containing DNAJB6b were stored on ice or frozen at −20 °C until used in biophysical experiments. The concentration of DNAJB6b was determined by mixing 150 µL from each fraction with 50 µL 8M GuHCl, recording the absorbance between 360 and 232 nm, multiplying the absorbance at 280 nm by 4/3, and dividing by the extinction coefficient 14,400 M^−1^cm^−1^.

### 4.5. Adaption of the Purification Protocol for Mutants

Variants with retained charge distribution, e.g., STA5, STA18, and T193A, can be purified using the same protocol as the wt. The purification of charge substitution mutants may require adjustment to the pH values of the buffers used. In such a case, a quick pH scouting with crude sonicates may be helpful to find out under which pH the variant is minimally soluble. When purifying the NCys and CCys mutants, it is possible to use the same protocol as for wt with the only modification being the inclusion of 1 mM DTT in all buffers to prevent the formation of covalent dimers.

### 4.6. Aggregation Kinetics

Aβ(M1-42), was expressed and purified as described [30] and monomers were isolated in 20 mM sodium phosphate, 0.2 mM EDTA, pH 8.0. For DNAJB6b wt, we used an aliquot from fraction 14 from the SEC run on Superos6 shown in Figure 3C,D. For DNAJB6b STA5 we used an aliquot from fraction 14 in the SEC run on Superos6 (Figure S1). Samples were kept and mixed on ice to contain 4 µM Aβ42 alone or 4 µM Aβ42 with DNAJB6b wt or STA5 at concentrations ranging from 2 to 150 nM. All samples contained 6 uM thioflavin T (ThT) and were distributed as multiple replicates in wells of a Corning 3881 96-well plate (a half area PEGylated black polystyrene plate with a transparent bottom). The plate was inserted in a plate reader (BMG omega) equilibrated at 37 °C. The fluorescence was read through the bottom of the plate using an excitation filter at 440 nM and an emission filter at 480 nm. The data were normalized using Amylofit [31] and could only be fitted using models assuming inhibition of primary nucleation.

### 4.7. Fluorophore Labelling

Purified DNAJB6-CCys or DNAJB6-NCys was subjected to SEC on a G25 column operated in 20 mM sodium phosphate, pH 8.0, to remove DTT. Alexa488, Alexa555, Alexa647, or IRdye680 was added from a 5 mM stock in DMSO, 1.5 molar equivalents. The solution was gently mixed and incubated at room temperature for 2 h. Excess dye was removed by SEC on a 10 × 300 mm Superdex75 column operated in 20 mM sodium phosphate, 0.2 mM EDTA, and pH 8.0.

## 5. Conclusions

We have presented here a versatile protocol for producing highly pure DNAJB6b wt and variants. The protocol relies on inexpensive tools as it is based on the physico-chemical properties of the protein, which is expressed as is with no sequence additions. The purified protein will thus be amenable to more detailed studies of these properties and their molecular origin using a range of biophysical tools including NMR and optical spectroscopy, as well as a multitude of surface and scattering techniques. The ease of isotope labeling will be an advantage both for NMR spectroscopy and neutron scattering with contrast variation to distinguish different components in co-aggregates. The palette of fluorescently labelled variants opens for additional experiments using fluorescence correlation spectroscopy, microscopy, and fluorescence resonance energy transfer. The plasmids described here will be shared with the research community and the protocols we describe for expression, purification, and labelling are easy to set up in any lab at low cost.

## Figures and Tables

**Table 1:** Expression and purification procedure and yield estimation.

Step	Amount of DNAJB6b per Litre Culture	Estimation Based on
Expression in *E. coli*	1500 mg/L	Gel band intensity
Sonication at pH 6.0, removal of soluble *E. coli* proteins and lightly precipitated ones.	1200 mg/L	Gel band intensity
Sonication at pH 8.0 followed by passage through anion and cation exchange resins	1200 mg/L	Gel band intensity
Ammonium sulphate precipitation to remove non-protein contaminants and some contaminating proteins	900 mg/L	Gel band intensity
Size exclusion chromatography to remove large and small contaminants (two steps)	600 mg/L	Absorbance in 2 M GuHCl

## Data Availability

All data will be shared with readers upon reasonable request.

## Supplementary Materials

The following supporting information can be downloaded online. Figure S1: Purification of DNAJB6b-STA5.

## Sample Availability

All plasmids will be shared with readers upon reasonable request.
