# Supplementary material for: High-Efficiency Expression and Purification of DNAJB6b Based on the pH-Modulation of Solubility and Denaturant-Modulation of Size
Source: Molecules. 2022 Jan 10;27(2):418. doi: 10.3390/molecules27020418 (PMC8781954; doi:10.3390/molecules27020418)
Supplement: Supplementary file 1 [file molecules-27-00418-s001.zip › molecules-1512116-supplementary.pdf]

High-efficiency expression and purification of DNAJB6b based on the pH-modulation of solubility and denaturant-modulation of size.

Sara Linse
Lund University, Biochemistry and Structural Biology, P O Box 124, Lund, Sweden.

Supplementary information

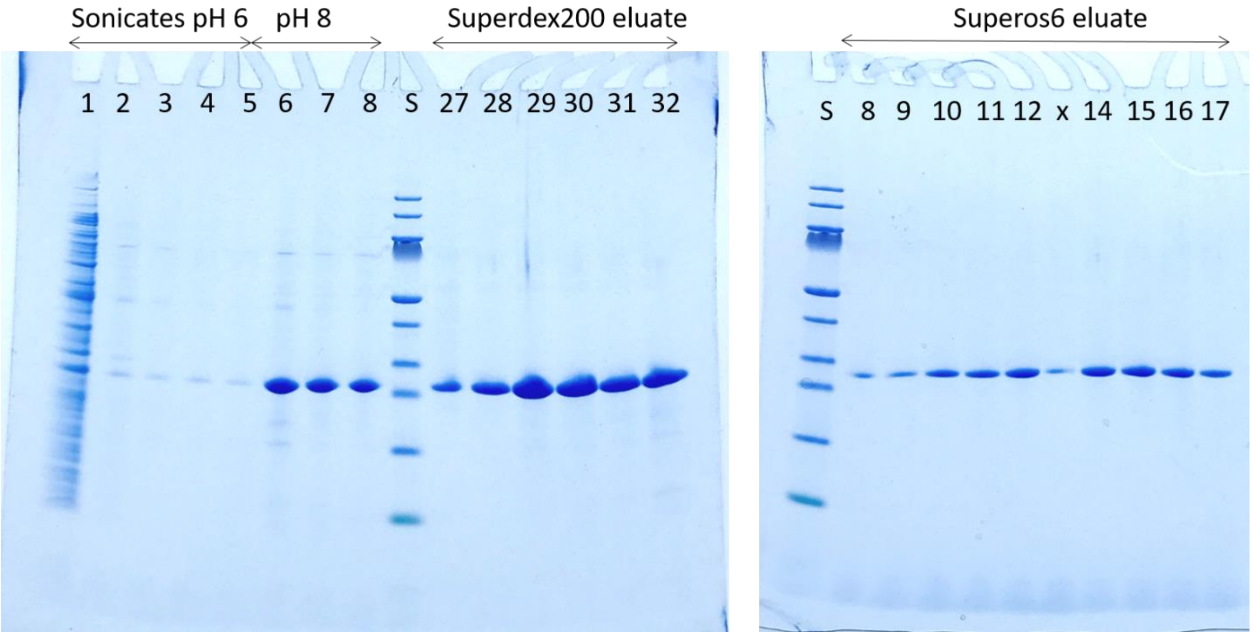

Figure S1. Purification of DNAJB6b-STA5. SDS PAGE on a 10-20% polyacrylamide gel with lane 1-5 loaded with sonicate 1-5, lane 6 with sonicate 6, lane 7 with the Q flow-through and lane 8 with the SP sepharose flow-through, lane S with M_w standard, same as used in Figures 2 and 3 of the main manuscript. The flow-through of SP sepharose (lane 8) was used for purification of the protein using ammonium sulphate precipitation and size-exclusion chromatography. An aliquot from the flowthrough of SP sepharose (see lane 8 in Figure 2) was precipitated by AMS and the 10-21% fraction dissolved in 10 mL 6 M GuHCl, 20 mM sodium phosphate, 0.2 mM EDTA, pH 8.0 and injected on a 26/600 Superdex200 column operated in 2 M GuHCl, 20 mM sodium phosphate, 0.2 mM EDTA, pH 8.0. Lanes 27-32 show fractions 27-32 of the eluate. Fraction collection started earlier than in the chromatogram shown in Figure 3A. Fraction 28-32 were concentrated to 6 M GuHCl and 5 mL injected on a 16/600 Superose6 column. The gel to the right shows fractions 8-12 and 14-17 from the elution of the 16/600 Superos6 column operated in 20 mM sodium phosphate, 0.2 mM EDTA, pH 8.0. By mistake fraction 13 was not loaded and lane x shows spillover from lane 12 or 14.
